# Impact of the First–Fourth Waves of the COVID-19 Pandemic on New Applications for Long-term Care Insurance in a Metropolitan Area of Japan

**DOI:** 10.2188/jea.JE20220084

**Published:** 2022-11-05

**Authors:** Satoshi Seino, Shoji Shinkai, Akihiko Kitamura, Yu Nofuji, Yuri Yokoyama, Toshiki Hata, Yoshinori Fujiwara

**Affiliations:** 1 Research Team for Social Participation and Community Health, Tokyo Metropolitan Institute of Gerontology, Tokyo, Japan; 2 Department of Nutrition Sciences, Kagawa Nutrition University, Saitama, Japan; 3 Health Town Development Science Center, Yao City Health Center, Osaka, Japan; 4 Integrated Research Initiative for Living Well with Dementia, Tokyo Metropolitan Institute of Gerontology, Tokyo, Japan

We previously reported that the number of new long-term care insurance (LTCI) applications^1^^,^^2^ in a metropolitan area of Japan decreased by 29.7–41.7% compared to predicted values during the first wave of the novel coronavirus disease 2019 (COVID-19) pandemic (March–May 2020).^3^^,^^4^ The number of LTCI applications jumped immediately after the first wave (June 2020).^3^^,^^4^ Although new LTCI applications seemed to be postponed during the first wave, it is unclear how the prolonged pandemic affected the number of new applications that followed. We now extend our report to address the impact through the fourth wave of the COVID-19 pandemic on new applications of LTCI in our metropolitan cohort.

As in the first report,^3^^,^^4^ we followed 15,500 stratified and randomly sampled residents aged 65–84 years for a community-wide intervention study in Ota City, Tokyo, in 2016.^5^ We investigated the applications and certifications for LTCI and calculated the number of new applications per 10,000 people every month from January 2018 to July 2021. Moreover, we obtained the number of new COVID-19 infections per day in Tokyo from the Ministry of Health, Labor, and Welfare’s open site.^6^ To the best of our knowledge, since there is no official definition of the term “waves,” we defined the months closest to the peak of each wave as the duration of the wave. Simple regression analysis using ordinary least squares, with the dependent variable as the number of new LTCI applications from January 2018 to February 2020 (pre-COVID-19 pandemic) and the independent variable as each month (a dummy form), was applied. The number of new LTCI applications after March 2020 (during the COVID-19 pandemic) was predicted using the regression equation. We compared the predicted and actual number of new LTCI applications from March 2020 to July 2021. This study was approved by the Ethical Committee of the Tokyo Metropolitan Institute of Gerontology (No. 22).

**Figure 1.**
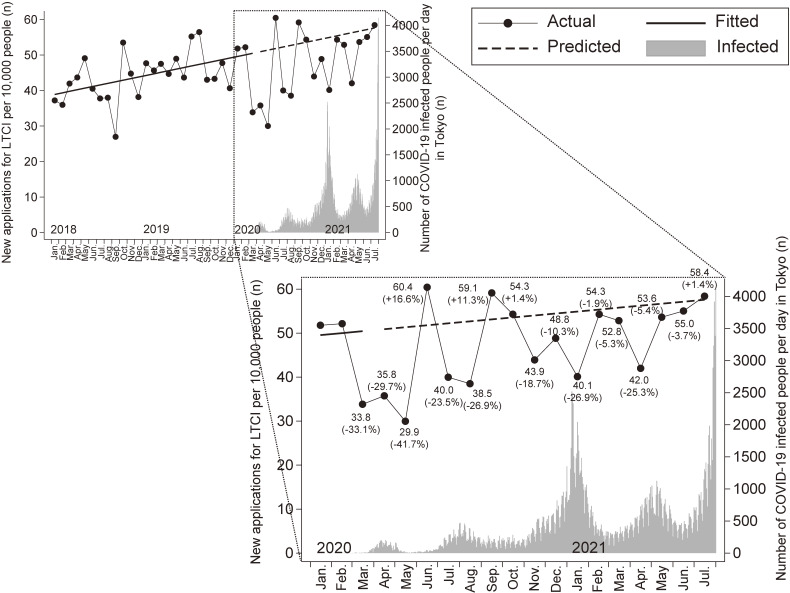
Number of new LTCI applications per 10,000 people monthly in Ota City cohort and the number of COVID-19 infected people per day in Tokyo. The solid line represents the regression line applied to the number of new LTCI applications from January 2018 to February 2020. The dotted line shows the predicted values based on regression. The grey bar shows the number of people infected with COVID-19 per day in Tokyo. The value in parentheses represents the percentage of the difference from the predicted value. COVID-19, novel coronavirus disease 2019; LTCI, long-term care insurance.

Figure 1 shows the number of new applications for LTCI per 10,000 people monthly in the Ota City cohort from January 2018 to July 2021 and the number of COVID-19 infected people per day in Tokyo through July 2021. In this study, March to May 2020 was defined as the first wave, July to August 2020 as the second wave, November 2020 to January 2021 as the third wave, April to May 2021 as the fourth wave, and July 2021 and beyond as the fifth wave. Compared with predicted values, the actual number of LTCI applications decreased by 23.5–26.9% in the second wave, 10.3–26.9% in the third wave, and 5.4–25.3% in the fourth wave, but was almost similar (+1.4%) to the predicted value at the beginning of the fifth wave. Although rebounds above the predicted values in the number of new LTCI applications were observed between the first and second waves (+16.6%, June 2020) and the second and third waves (+1.4–11.3%, September to October 2020), no such rebound tendencies were observed between the third and fourth waves (−1.9 to −5.3%, February to March 2021) or between the fourth and the beginning of the fifth waves (−3.7%, June 2021).

There was a clear trade-off between increased numbers of COVID-19 cases during the first to fourth waves of the COVID-19 pandemic and decreased numbers of LCTI applications. However, the rebound in the number of new LTCI applications observed after the first to fourth waves suggests the temporary postponement of new LTCI applications. The number of new LTCI applications during the pandemic did not seem to increase compared with that before the pandemic. Instead, the number of new LTCI applications since March 2020 tended to be generally suppressed compared with the predicted value, suggesting that older adults and their families were refraining from new LTCI applications. It should be noted that the number of outcomes regarding LTCI is generally underestimated until the fourth wave of the pandemic in our cohort.

There are several possible explanations for this. Many older adults and their families might have been refrained from LTCI applications due to concerns about the increase in the number of new infections and the emergence of the strains in each wave. Perhaps, staying home and working from home during the pandemic might have allowed the older adults’ families to support older adults. Impressively, as the number of waves has increased, the degree of decrease in the number of new LTCI applications has decreased; this trade-off was not observed at the beginning of the fifth wave. The number of new LTCI applications is returning to the pre-pandemic level, possibly owning to the impact of increasing vaccination prevalence in the older population and experience with the pandemic. The number of new LTCI applications and classification changes may increase in the future due to the influence of a long-term suppression of physical activity^7^ and social interaction.^8^ Alternatively, these may start to decrease again, if a new strain of COVID-19 emerges that is highly infectious and with a high risk of aggravation. Therefore, we still need to monitor the number of these applications.
